# Income diversification and liquidity risk in ASEAN-5 banks: A Bayesian perspective

**DOI:** 10.1371/journal.pone.0316949

**Published:** 2025-03-05

**Authors:** Quynh Nga Duong, Nguyen Thuy Khue Tran, Thi Phuong Thao Dang

**Affiliations:** 1 Faculty of Air Transport Economics, Vietnam Aviation Academy, Ho Chi Minh City, Vietnam; 2 Faculty of Business Administration, Vietnam Aviation Academy, Ho Chi Minh City, Vietnam; University of Rome Tor Vergata: Universita degli Studi di Roma Tor Vergata, ITALY

## Abstract

Our research employed Bayesian linear regression utilizing an adaptive Metropolis-Hastings method with Gibbs sampling to assess the influence of bank income diversification on the liquidity risk of five ASEAN banks. The results indicate a positive relationship between bank liquidity risk and income diversification, as well as loan interest rates. This implies that banks with greater income diversification tend to have higher liquidity ratios and reduce the bank risk and conversely. Therefore, the study suggests that banks should enhance their diversification efforts to mitigate their liquidity risk

## Introduction

Income diversification impacts liquidity creation in banks by providing benefits such as increased income streams, economies of scale, reduced volatility, and lower insolvency risks [1]. Traditional banking, which connects core deposits with relationship loans, tends to create more liquidity compared to non-traditional activities like brokerage and underwriting [2]. Income diversification often correlates with higher liquidity creation, indicating a positive relationship between the two. The financial sector’s role in economic growth, through liquidity creation, is crucial for stability and development. Diversifying income sources helps banks withstand market volatility and financial shocks, enhancing financial stability. Research on the impact of income on bank liquidity in developing economies remains limited, despite regulations aimed at strengthening the banking sector.. Past studies have focused on developed economies, which may not be applicable to developing countries due to cultural and regulatory differences [1–4].

ASEAN nations frequently face economic instability due to factors such as political unrest, changes in commodity prices, and global economic fluctuations. To counter these risks, banks in these regions diversify their income sources, reducing reliance on traditional interest-based revenue. This approach is vital for maintaining stability during economic downturns [5–7]. Given the history of financial crises in the region, such as the 1997-1998 Asian Financial Crisis, financial stability is a critical priority. Income diversification strengthens banks’ resilience, allowing them to better endure shocks and maintain effective operations, which is essential for sustainable economic growth in ASEAN [8,9].

Diversifying into new revenue streams, such as brokerage fees, underwriting, and other non-traditional banking activities, is crucial for improving profitability, especially in a low-interest-rate environment where traditional banking margins are under pressure. Research indicates that banks with diversified income sources generally achieve better financial performance and higher firm value [4,10].

Moreover, advancements in financial technology offer ASEAN banks new opportunities to diversify their services. By adopting digital banking platforms, collaborating with fintech companies, and offering investment products, banks can enhance their non-interest income streams. This technological adaptation is essential for remaining competitive in the rapidly evolving financial sector [2,11].

Income diversification also provides ASEAN banks with a competitive advantage by enabling them to offer a wider range of services to their customers. This not only helps attract new clients but also aids in retaining existing ones by addressing diverse financial needs. The ability to innovate and provide comprehensive financial solutions positions these banks favorably in a highly competitive market [12].

Many authors have conducted research on the factors affecting bank liquidity and diversification in this area, yielding certain results. Although each study focuses on different factors, they all influence the liquidity of commercial banks. However, there has been limited research specifically examining diversification as the main factor impacting liquidity.

Despite notable progress in understanding the role of income diversification in banking performance, there remains a significant gap in the literature regarding its specific effects on the liquidity ratios of banks in the ASEAN region. While many studies have examined how income diversification—primarily through non-interest income—affects bank profitability, risk, and stability, far fewer have addressed its influence on banks’ liquidity positions. Most of the existing research on this topic centers on developed economies or non-ASEAN developing nations. However, the ASEAN banking sector possesses unique characteristics, such as diverse regulatory frameworks, varying levels of financial development, and a greater reliance on traditional banking practices, which may lead to different liquidity dynamics compared to other regions. This highlights the need for more focused research on ASEAN banks.

In recent years, the ASEAN banking sector has experienced significant changes, including regulatory reforms like the implementation of Basel III, greater economic integration, and advances in technology. These shifts could alter the traditional relationship between income diversification and liquidity, emphasizing the need for updated empirical studies that reflect the current environment. Previous research has largely focused on overall income diversification without sufficiently distinguishing between specific non-interest income sources such as fee-based income, trading income, and other non-traditional revenue streams. The impact of these individual income channels on liquidity ratios (e.g., liquid assets to total assets, loan-to-deposit ratio) in ASEAN banks remains insufficiently explored.

This study addresses this gap by being the first to thoroughly analyze the impact of income diversification on liquidity risk in ASEAN-5 countries. It should also consider the moderating effects of regulatory frameworks and macroeconomic conditions while using more recent and comprehensive data that reflect the current financial landscape. Understanding this relationship is vital for policymakers, regulators, and banking institutions as they navigate the challenges of financial stability and economic growth in the ASEAN region. This research is crucial as it provides guidance for managers and stakeholders, particularly in comparable emerging economies, on strategies to enhance commercial banks’ liquidity creation through capital requirements and diversification.

The paper is structured as follows: Section 2 offers a literature review and hypothesis development, setting the foundation for understanding the relationship between income diversification and liquidity risk. Section 3 outlines the data and methodology used in the study and details the analytical techniques and data sources. Section 4 presents the empirical findings and discussions, providing an in-depth analysis of the results and their implications for the banking sector. Finally, Section 5 concludes the paper with policy implications, offering recommendations for improving liquidity ratios in commercial banks through strategic diversification and regulatory measures.

## Literature review and hypotheses development

The relationship between liquidity ratios and revenue diversification is complex and varies across different sectors and organizational structures. The interaction between these financial aspects can significantly affect a company’s financial strategy and overall performance. This analysis will examine how diversification influences liquidity, based on insights from relevant research studies.

By broadening the revenue base and minimizing reliance on a single income source, income diversification contributes to greater financial stability in banks. This stability is vital for maintaining healthy liquidity ratios, as it ensures banks have sufficient liquid assets to meet short-term obligations [13]. Research on Chinese banks indicates that income diversification is more advantageous than asset diversification in terms of profitability and risk management, indirectly supporting liquidity by stabilizing income flows [14].

Within the ASEAN Economic Community (AEC), integration drives banks to adopt diversification strategies to boost performance, positively affecting liquidity ratios by enhancing financial resilience [10]. Additionally, trade diversification, as part of wider economic strategies, promotes economic growth and stability, which benefits the banking sector’s liquidity by fostering a stronger economic environment [15]. Toh et al. (2020) found that income diversification positively influenced bank liquidity creation in Malaysian banks, enhancing their resilience [7]. Similarly, Hou et al. (2018) observed a positive relationship between diversification and liquidity creation in non-traditional banking activities in China [4].

Additional research highlights that income diversification can maximize profits, achieve economies of scale, reduce volatility, and lower insolvency risks [4]. Portfolio theory supports that income diversification reduces idiosyncratic and overall risks [5], enhancing the risk-return frontier and long-term financial performance [4].

Casu et al. (2019) emphasize that risk-reduction benefits from diversification strengthen banks’ foundations, improving liquidity creation capabilities essential for financial stability and economic growth [12]. Diversifying income sources is vital for banks’ strategic management, helping to enhance financial stability and liquidity capacity.

Despite mixed findings, the general consensus is that income diversification offers significant benefits, aiding in risk management and supporting liquidity creation in competitive and volatile environments. This has become more important as banks face increasing competition and regulatory pressures, prompting a shift from traditional interest-based activities to non-interest activities [11,12]. Research has demonstrated that income diversification enhances bank performance, firm value, and stock prices, but its influence on risk is still intricate.

Traditional theories, such as Markowitz’s portfolio theory (1952) and economies of scope, argue that income diversification lowers risk. Banks can earn low-risk income from new activities based on information gained from traditional operations, mitigating information asymmetry [6].

By leveraging information from traditional operations, banks can generate low-risk income through new activities, thereby reducing information asymmetry.. This helps smooth revenue volatility and achieve stable financial performance, enabling banks to innovate and stay competitive.

Mu’izzuddin and Isnurhadi (2024) examined the effects of business cycles and income diversification on capital buffers and bank risk in ASEAN from 2020 to 2022. They found that diversified revenue streams could weaken capital buffers but reduce overall risk, indicating a nuanced relationship between diversification and liquidity creation.

Overall, income diversification is seen as beneficial for enhancing bank performance, firm value, and stock prices, providing a solid theoretical foundation for mitigating risks and supporting long-term stability and growth. Empirical studies support that diversification can reduce risk, though some research highlights potential downsides, such as agency problems and increased income volatility.

Limited research on banking liquidity in Southeast Asia highlights a gap in the literature, with most studies focusing on developed nations. Existing research in Southeast Asia primarily addresses other aspects like profitability determinants, liquidity and market risk, and the impact of liquidity risk on bank performance.This paper aims to fill this research gap by examining the determinants of commercial banks’ liquidity in Southeast Asia, specifically in Malaysia, Indonesia, the Philippines, Thailand, and Vietnam.

The primary research question addressed is: “What impact does income diversification have on liquidity risk?”

We propose the following hypothesis as a solution to this question:

H1: Income diversification has a positive impact on banks’ liquidity ratios that reduce a banks’ liquidity risk.

## Data and methodology

### Data

This study utilized a panel dataset of all commercial banks operating in the ASEAN-5 countries from 2007 to 2020. The primary data source was the financial reports published by these commercial banks. As of December 2022, there were 53 commercial banks in the ASEAN-5 countries. Due to insufficient data, six commercial banks were excluded, reducing the sample size to 35 commercial banks. We used this process to create an unbalanced panel dataset for analysis.

This study employed the Bayesian approach to test the hypothesis because it allows for the incorporation of prior information and uncertainty in parameter estimates, which is particularly useful when data is limited or the model involves complex relationships. The Bayesian method provides a flexible framework for updating beliefs based on new data and offers probabilistic interpretations of model parameters. This approach is well-suited for evaluating bank performance, risk, and diversification, as it can accommodate various sources of uncertainty and provide more robust estimates compared to traditional frequentist methods. Moreover, Bayesian techniques are advantageous in handling hierarchical models and can effectively incorporate data from multiple sources, such as macroeconomic factors and bank-specific characteristics.

### Empirical research model

Based on the theoretical foundation and the hypotheses developed above, as well as building on the prior studies, we develop a research model examining the relationship between income diversification and bank diversification, with control variables representing bank-specific characteristics.

Model 1:


$$LC1_{it}=\alpha_{0}+\beta_{1}LC\_CF_{it-1}+\beta_{2}DIV_{it}+\beta_{2}NLTA_{it}+\beta_{3}SIZE_{it}+\beta_{4}ASSET\_GROW_{it}+\beta_{2}GDP_{it}+\beta_{2}INF_{it}+\phi_{i}+\upsilon_{t}+\varepsilon_{it}$$

Model 2:


$$LC2_{it}=\alpha_{0}+\beta_{1}LC\_CNF_{it-1}+\beta_{2}DIV_{it}+\beta_{2}NLTA_{it}+\beta_{3}SIZE_{it}+\beta_{4}ASSET\_GROW_{it}+\beta_{2}GDP_{it}+\beta_{2}INF_{it}+\phi_{i}+\upsilon_{t}+\varepsilon_{it}$$

$LC_{it}$ and $LC_{it-1}$: the current and previous years’ liquidity created by commercial banks. $DIV_{it}$ and $NLTA_{it}$ represent the independent variables (income diversification and net loans to assets ratio) and control variables (equity to total assets EQTA, bank size SIZE, asset growth ASET_GROW, gross domestic product GDP and inflation INF). $\phi_{i}$, $\upsilon_{t}$ and $\varepsilon_{it}$ represent unobserved bank-specific effects, temporal dummy and the error term, respectively. The temporal dummy was responsible for the specific effect of time. *i* and *t* represent the period and individual bank, respectively.

### Measurement of study variables

#### Liquidity risk.

Liquidity ratios are various balance sheet ratios designed to identify key liquidity trends. These ratios ensure that banks can access appropriate, low-cost funding quickly. This can be achieved by maintaining a portfolio of easily sellable assets (such as cash reserves, required minimum reserves, or government securities), holding significant volumes of stable liabilities (especially retail deposits), or maintaining credit lines with other financial institutions.

Liquidity risk encompasses two types of risk: funding liquidity risk and market liquidity risk. Funding liquidity risk is the possibility that a bank cannot efficiently meet both expected and unexpected current and future cash flow and collateral needs without adversely affecting daily operations or financial health. Market liquidity risk is the risk that a bank cannot easily offset or eliminate a position at the market price due to insufficient market depth or market disruption.

Liquidity risk can be assessed using two primary methods: liquidity gaps and liquidity ratios. The liquidity gap measures the difference between assets and liabilities at present and future dates, where a positive gap indicates a deficit at any given date.

Several authors, such as Praet (2008), and Rychtárik (2009), have explored various liquidity ratios. For the purposes of this research, the following liquidity ratios will be utilized:


$$LC1=\frac{liquidassets}{totalassets}\times 100\%$$

The liquidity ratio LC1 should give us information about the general liquidity shock absorption capacity of the bank. As a general rule, the higher the share of liquid assets in total assets, the higher the capacity to absorb liquidity shock, given that market liquidity is the same for all banks in the sample. However, a high ratio could potentially indicate inefficiency, as liquid assets reduce income liquidity and impose significant opportunity costs on the bank. Thus it is necessary to optimize the relation between liquidity and profitability.


$$LC2=\frac{totalloans}{totalassets}\times 100\%$$

The ratio LC2 measures the share of loans in total assets. It indicates what percentage of the assets of the bank is tied up in illiquid loans. Therefore, the higher this ratio, the less liquid the bank is.

#### Income diversification.

We measure income diversification using the traditional Herfindahl-Hirschman index. The share of non-interest income is calculated as the ratio of non-interest income to total operating income, following the common approach in the existing literature [5,11]. A higher ratio indicates greater bank involvement in non-traditional activities. This measure is based on the diversification approach, where larger values indicate a higher degree of income diversification. The income diversification measure, as defined by the Herfindahl-Hirschman index, is referenced from studies by Doan et al. (2018) and Elsas et al. (2010):


$$DIV=1-HHI=1-[{\left(non-interestincomeshare\right)}^{2}+{\left(interestincomeshares\right)}^{2}$$

Banks with income diversification strategies are expected to generate higher income on sources beyond traditional banking activities (interest operations). Therefore, a higher DIV implies a higher level of bank diversification.

#### Other control variables.

Equity to Total Asset Ratio (EQTA): is calculated as total equity divided by total assets. The debt to total assets ratio is an important solvency measure that shows the percentage of a bank’s assets financed by debt. It provides insight into the bank’s ability to manage its debt in relation to its asset base, which is crucial for assessing its overall financial stability and solvency.

Bank Size (SIZE) is one of the most frequently considered bank-specific determinants and is highlighted by Kumar et al. (2022) as a key driver of banks’ profitability. Larger banks are generally expected to be more profitable due to economies of scale. These banks have better access to a wider range of funding sources and more sophisticated cost management techniques to diversify their portfolios. Conversely, smaller banks face higher costs and are expected to be less profitable.

In addition to bank-specific profitability determinants, researchers also consider macroeconomic factors such as inflation and GDP growth. The macroeconomic environment can significantly impact banks’ behavior and performance. Depending on the period, geographic location, and model used, these determinants may vary across studies.

The growth of assets (ASSET_GROW) may indicate a high risk appetite of the management. The rate of real GDP growth (GDP) measures the development level of an economy. Inflation (INF) is typically calculated through the consumer price index. Since mid-2022, inflation in Asia has moderated due to declines in commodity prices and shipping costs. However, the primary driver of headline inflation in Asia has shifted toward rising core inflation, similar to global trends. Core inflation has remained above central bank targets in most Asian economies, though it is still moderate compared to the rest of the world.

### Empirical findings and discussions

#### Descriptive statistics.

The research involved 34 banks from five ASEAN countries that operated during the observed period. The study excluded only one bank that had operated for less than five years. This resulted in a dataset comprising 490 observations. Table 1 provides descriptive statistics on the chosen dependent and independent variables for banks in the ASEAN-5 region from 2008 to 2022.

**Table 1:** Variable Measures Summary.

Dependent variables	Variable	Notation	Expected effect
	Liquidity risk 1	LC1	
	Liquidity risk 2	LC2	
Indepent variables	Liquidity risk 1 lag 1	LC1_lag1	Positive
	Liquidity risk 2 lag 1	LC2_lag1	Positive
	Income diversification	DIV	Positive
Bank specific control variables	Equity to total asset	EQTA	Negative
	Bank size	SIZE	Positive
Macroeconomic control variables	Real Growth Rate of Gross Domestic Product	GDP	Positive
	Inflation rate	INF	Negative/positive

For the dependent variable (LC1) and (LC2), the means of LC1 and LC2 are 0.4 and 0.3, respectively. Besides, the standard deviation is 0.17 and 0.11. Which explains a huge difference in the scope of the liquidity risk between these Southeast Asian countries. For the main independent variable (DIV) have an average of 0.84 by standard deviation of 0.09 which alludes a small deviation in income diversification in five ASEAN countries. For bank size (size), we find a mean of 4.18 by standard deviation of 0.56.

The mean value of income diversification (DIV) is 0.84 (SD =  0.09). Given that the maximum value of this indicator is 0.94, the obtained mean value of this variable indicates a relatively high degree of net income diversification. Standard deviation points to the similarity of the applied income diversification strategies of the banks in the sample. DIV does not show whether banks are more focused on interest or non-interest activities.

#### Stationarity test.

The validity of our results depends on the convergence of the MCMC process. To assess convergence, we visually examine the trace plot of liquidity risk, which shows good mixing. The autocorrelation diminishes rapidly, and the posterior distribution aligns with the normal distribution, consistent with the specified likelihood and prior distributions. Thus, there is no indication of non-convergence.

Our results are valid only if the MCMC process has converged. To assess this, we evaluate the convergence of MCMC using $R_{C}$ and ESS values. Tables 2 and 3 show that the maximum $R_{C}$ value from the Gelman-Rubin diagnostics is 1.00034, which is less than 1.1, and the smallest ESS is 0.8571. Therefore, we conclude that the MCMC has successfully converged.

**Table 2:** Descriptive statistics summary.

Variable	Obs	Mean	Std. Dev	Min	Max
LC1	490	0.4370	0.1739	0.0099	1.4233
LC2	490	0.3325	0.1169	0.0011	0.6855
LC1_lag1	455	0.4368	-.1767	0.0099	1.4233
LC2_lag1	455	0.3314	0.1173	0.0011	0.6855
DIV	490	0.8412	0.0971	0.6179	1.2230
SIZE	490	4.1876	.05642	2.6341	5.3294
EQTA	490	0.0327	0.0585	0.00005	0.6193
ASSET_GRO	490	0.0495	0.0757	-0.1832	0.5134
GDP	490	4.5203	3.0595	-9.573	8.464
INF	490	4.0080	3.6651	-1.139	23.114

(Source: Author’s calculations).

**Table 3:** MCMC convergence of model 1 and model 2.

	$R_{C}$ Model 1	$R_{C}$ Model 2
LC_lag1	1.0001	1.0001
DIV	1.0000	0.9999
EQTA	1.0000	1.0001
SIZE	1.0003	1.0000
ASSET_GRO	0.9999	0.9999
GDP	1.0000	1.0000
INF	1.0000	0.9999
Cons_	1.0000	1.0014

(Source: Author’s calculations).

#### Multicollinearity test/correlation analysis.

The correlation coefficient is commonly used measure to determine the association between two random variables. Significant focus has been placed on interpreting this coefficient and developing methods to correct for attenuation caused by random measurement error. The traditional methods solely rely on the collected data, disregarding any prior knowledge about the association being studied. An alternative approach is the Bayesian method, which incorporates knowledge from previous studies to enhance the estimation of correlation coefficients.

#### The effect of income diversification on bank liquidity risks.

To report preliminary estimates, we exhibit the posterior mean of the parameters and a 95% credible interval, which contains the parameters of interest with a certain probability in Tables 4 and 5. If a particular parameter has a positive (negative) posterior mean and the probability of its positive (negative) effect in the 95% credible interval is greater than 50%, it is rated to cause a strongly positive (strongly negative) impact.

**Table 4:** Bayesian estimation results of model 1.

Independent variables	Posterior mean	MCSE	Probability of mean (%)	ESS	Equal-tailed [95% cred. Interval]	
LC1_lag1	0.8039	0.0012	95	29518	0.7189	0.8253
DIV	0.0031	0.0012	94.9	30000	-0.1073	0.1132
EQTA	0.0213	0.0057	93.7	30000	-1.9424	1.9653
SIZE	0.0001	0.0012	94.9	30000	-0.0205	0.0205
ASSET_GRO	-0.2872	0.0012	95	30000	-0.4183	-0.1570
GDP	0.0010	0.0013	94.6	29786	-0.0013	0.0048
INF	-0.0103	0.0012	94.6	30000	-0.0037	0.0016
Cons	-17.2531	0.0012	94.9	30000	-0.0550	0.2650

(Source: Author’s calculations).

**Table 5:** Bayesian estimation results of model 2.

Independent variables	Posterior mean	MCSE	Probability of mean (%)	ESS	Equal-tailed [95% cred. Interval]	
LC2_lag1	0.8562	0.0015	8.1	30000	0.8128	0.8995
DIV	-0.0276	0.0012	94.98	30000	-0.0890	0.0329
EQTA	0.1282	0.0057	92.5	30000	-1.8397	2.0757
SIZE	-0.0046	0.0012	94.8	30000	-0.1609	0.0067
ASSET_GRO	-0.1699	0.0012	95	30000	-0.2436	-0.0961
GDP	0.0023	0.0012	94.7	30000	0.0006	0.0040
INF	-0.0004	0.0000	0	30000	-0.0019	0.0010
Cons	0.0926	0.0012	94.9	30000	0.0050	0.1815

(Source: Author’s calculations).

Tables 4 and 5 present the panel Bayesian regression estimation results on the effect of bank diversification on liquidity risks. Two tables report an estimation table that includes the posterior mean, MCMC standard error (MCSE), probability of mean, ESS and 95% credible interval.

An overall acceptance rate (AR) of model 1 and model 2 is also, meaning that 100% out of 10,000 proposal parameter values were accepted by the algorithm. This represents an excellent AR for the MH algorithm.

The estimated posterior mean for LC1_lag1 is 0.8 with a posterior standard deviation of 0.03. The efficiency of the estimator of the posterior mean is about 98.39%, which is very high for the random walk MH sampling. According to the reported 95% credible interval, the probability that the mean of LC in this model is between 0.7 and 0.8. The result indicates that liquidity of risk of previous year increases the liquidity of risk of the current year. The estimated posterior mean for LC2_lag1 is 0.8 with a posterior standard deviation of 0.02. the efficiency of the estimator of the posterior mean is about 8.1%, which is low for the random walk MH sampling. According to the reported 95% credible interval, the probability that the mean of LC2 in this model is between 0.81 and 0.89. The result indicates that liquility of risk of previous year increase the liquility of risk of the current year. The increasing of LC2, could make the bank become more illiquid, meaning that the bank will find it difficult to fulfill its short-term liabilities, such as sudden customer deposit withdrawals.

The closer the ESS estimates are to the MCMC sample size, the better. Additionally, the lower the correlation times are and the higher the efficiencies are, the better. Our results show that ESS is closer for the MCMC sample size for all variables.

The estimated posterior mean for DIV is 0.003 in model 1 and -0.027 with a posterior standard deviation of 0.05 and 0.03. The efficiency of the estimator of the posterior mean is about 94.9%, which is very high for the random walk MH sampling. According to the reported 95% credible interval, the probability that the mean of DIV in this model is between -0.1 and 0.1 and -0.8 and 0.03. The result indicates that income diversification increases the liquidity of risk in model 1 but decreases the liquidity risk in model 2. This finding implies that ASEAN – 5 banks with more diversified income has a higher liquidity risk ratio 1 and have a negative impact on liquidity risk ratio 2. The liquidity ratio LC1 give us information about the general liquidity shock absorption capacity of the bank. As a general rule, the higher the share of liquid assets in total assets, the higher the capacity to absorb liquidity shock, given that market liquidity is the same for all banks in the sample. High value of this liquidity ratio LC1 may also be interpreted as inefficiency, since liquid assets lower income liquidity bears high opportunity costs for the bank. Our results of the model are concordant with the results of Hou et al. (2018) but contrary to the results of Berger et al. (2017), Meslier (2019), Milbourn et al. (1999), and Aggarwal and Samwick (2003). They have identified the negative effects of bank diversification, while model 2 demonstrates the opposite effect. As banks diversify more, they generate higher profits, allowing them to cover expenses or settle debts. Additionally, an increase in post-tax profits enhances the bank’s reputation, which boosts depositor confidence and enables the bank to attract substantial capital. This, in turn, helps the bank stabilize liquidity by investing in liquid assets.

In the case of the control variable, we find that equity to total assets (EQTA) and GDP growth (GDP) have a positive and strong effect on the liquidity risk in 2 models. GDP growth is positively associated with the growth rate of bank liquidity creation, suggesting that bank liquidity creation is procyclical [15]. This correlation indicates that higher inflation rates lead to decreased bank liquidity, and vice versa. When inflation rises, the capital mobilization market encounters challenges, and people may withdraw their money from banks to invest in other, higher-return channels. Some commercial banks with an advantage in capital mobilization have opted to lend on the interbank market at high-interest rates, which carries lower risk than customer lending. As a result, the banking system circulates capital within itself instead of lending it out to the broader economy, thereby increasing the overall risk in the Vietnamese banking sector. In recent years, the State Bank has implemented various measures to control inflation, which has, in turn, improved bank liquidity. Bank liquidity generally rises with economic growth and decreases during economic downturns. This can be attributed to several factors: as the economy grows, people’s savings tend to increase, leading to greater capital mobilization. Banks can then invest more in liquid assets by purchasing short-term business securities, making deposits, and lending to other credit institutions, which enhances their liquidity. Additionally, economic growth increases businesses’ capital needs. If banks implement a sound credit growth policy, they can effectively utilize their liquidity to boost profits, thereby maintaining and stabilizing their liquidity.

Bank size (Size) and asset growth (ASSET_GRO) are strongly positive in model 1 but strongly negative in model 2. Our result is concordant with the study of Niu (2022). This is contrary to result found by Malik and Rafique(2013). Kashyapet al. (2002) find a strong effect of bank size on theholding of liquid assets, with smaller banks being more liquid as they face constraints in accessing capital markets. The proportional relationship suggests that as a bank grows in size, its liquidity also rises. This is because liquid assets typically make up a significant portion of a bank’s total assets. Thus, when the total assets increase, liquidity generally increases as well. An increase in this index (asset growth) indicates that the bank’s assets are growing and that there is less liquidity risk in the bank’s operations. Moreover, stable assets instill confidence in customers, making them feel secure and allowing the bank to easily mobilize large amounts of capital when necessary. A stable capital structure enhances the bank’s market reputation, enabling it to attract significant capital. However, as the bank reaches a certain level of capital, it may recognize its strength and potential for further development and start investing in liquid assets. This approach helps the bank create an efficient capital structure.

Inflation (INF) has a strong negative effect on model 1 and has a weak negative effect on model 2. A high inflation rate reduces borrowers’ ability to repay, leading to a significant increase in non-performing loans, which in turn decreases the liquidity risk ratio.

## Conclusion and policy implications

This study examines the effect of income diversification on the liquidity creation of commercial banks in ASEAN-5. The results show that increased income diversification significantly reduces bank liquidity risk, indicating that well-diversified banks have lower levels of liquidity risk. This supports portfolio theory and the theory of economies of scope under the synergy effect. Additionally, the study found a significant negative relationship between macroeconomic conditions and liquidity risk, suggesting that higher GDP levels reduce liquidity risk.

These findings imply that commercial banks should focus on both interest and non-interest income sources. Bank managers should prioritize income diversification to create new revenue streams that can buffer against financial shocks and maximize profits. Income diversification is crucial for enabling banks to withstand market volatility and financial instability. Therefore, commercial banks should adopt income diversification strategies as a risk management tool. By promoting a diversified income base, banks can avoid reliance on a single income source, thereby enhancing their liquidity resilience and stability. The inverse relationship between the equity to total assets ratio (EQTA) and bank liquidity can be explained as follows: the equity to total assets ratio (EQTA) measures a bank’s financial leverage, indicating the proportion of a bank’s assets financed by its own equity rather than debt. A higher EQTA means the bank relies more on equity to fund its assets, reflecting lower leverage and higher solvency. While a higher EQTA ratio strengthens a bank’s solvency, it can have an inverse effect on liquidity. A higher EQTA means the bank is using less debt to finance its operations. Debt, especially short-term borrowing, is a major source of liquidity for banks. When a bank reduces its debt, it may also reduce its liquid resources, as debt financing typically offers immediate funds for covering liquidity needs. Equity capital is typically less liquid compared to borrowed funds. The purpose of equity is to serve as a long-term financial buffer rather than an immediate cash pool. As a result, when a bank increases its reliance on equity, it may have fewer short-term liquid assets available to meet immediate liabilities or customer withdrawals. The inverse relationship between the EQTA ratio and bank liquidity arises because as a bank increases its equity relative to total assets, it reduces its reliance on debt and short-term funding, which are often key sources of liquidity. Additionally, equity is typically less liquid and more expensive than debt, leading banks with high EQTA ratios to maintain lower liquidity levels. While this improves a bank’s solvency, it can reduce its immediate liquidity, creating an inverse relationship between the two.

Develop a skilled workforce capable of mastering new technologies, along with professional marketing staff, to effectively promote banking products and services and enhance employee professionalism and transaction culture.

State banks can play a crucial role in enhancing the impact of income diversification on the liquidity ratios of ASEAN commercial banks by implementing strategic measures. These include promoting non-interest income sources such as wealth management and fee-based services, encouraging the development of innovative financial products through collaborations with FinTech, and simplifying regulatory hurdles to support diversified revenue generation.

State banks should also provide financial support and technical assistance to help commercial banks adopt advanced liquidity management tools and stress-testing mechanisms, ensuring diversified income streams positively influence liquidity. Encouraging regional cooperation among ASEAN banks for knowledge sharing and fostering cross-border financial products can further enhance income diversification efforts.

Additionally, state banks should drive digital transformation and support sustainability-linked financial products like green bonds and ESG initiatives. These steps will help banks generate stable income, improve liquidity ratios, and create a more resilient banking sector in the region.

## Supporting information

S1 Table Variable Measures Summary. (TIF)

S2 Table Descriptive statistics summary. (TIF)

S3 Table MCMC convergence of model 1 and model 2. (TIF)

S4 Table Bayesian estimation results of model 1. (TIF)

S5 Table Bayesian estimation results of model 2. (TIF)
