# Supplementary figures and images for: Income diversification and liquidity risk in ASEAN-5 banks: A Bayesian perspective
Source: PLoS One. 2025 Mar 5;20(3):e0316949. doi: 10.1371/journal.pone.0316949 (PMC11882047; doi:10.1371/journal.pone.0316949)

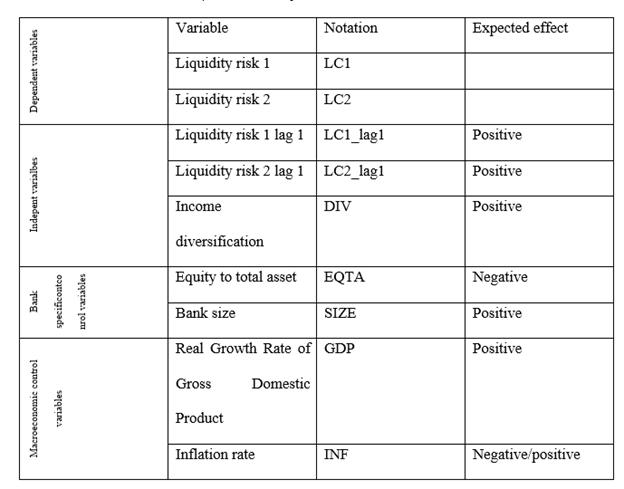

Supplement: S1 Table — (TIF) [file pone.0316949.s001.tif]

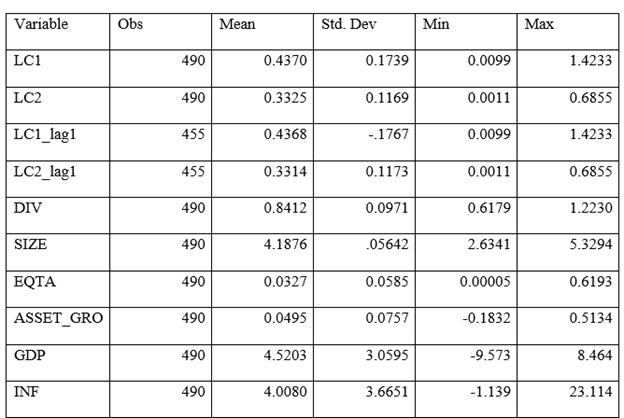

Supplement: S2 Table — (TIF) [file pone.0316949.s002.tif]

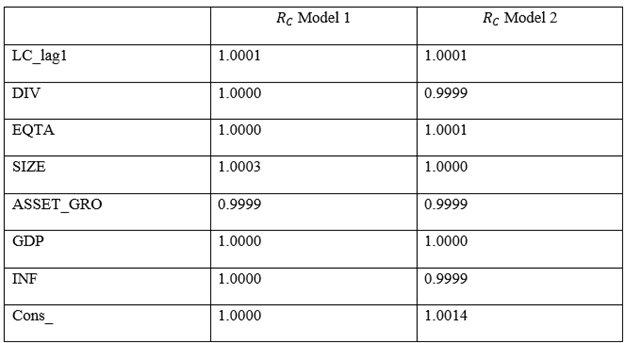

Supplement: S3 Table — (TIF) [file pone.0316949.s003.tif]

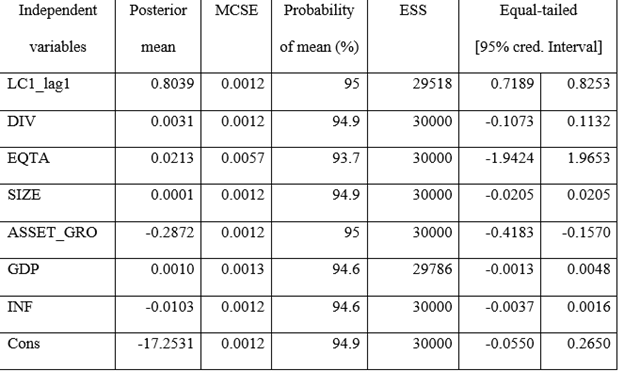

Supplement: S4 Table — (TIF) [file pone.0316949.s004.tif]

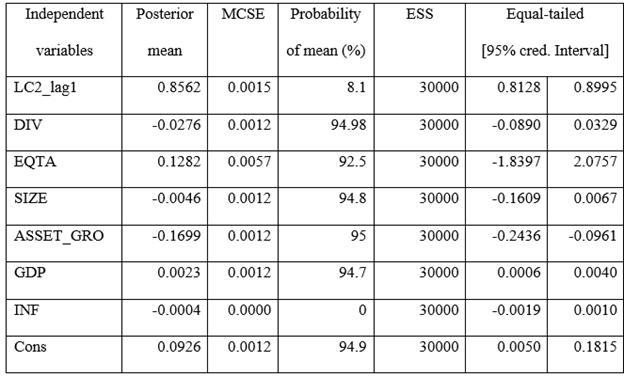

Supplement: S5 Table — (TIF) [file pone.0316949.s005.tif]
